# Supplementary figures and images for: Tramtrack acts during late pupal development to direct ant caste identity
Source: PLoS Genet. 2021 Sep 22;17(9):e1009801. doi: 10.1371/journal.pgen.1009801 (PMC8489709; doi:10.1371/journal.pgen.1009801)

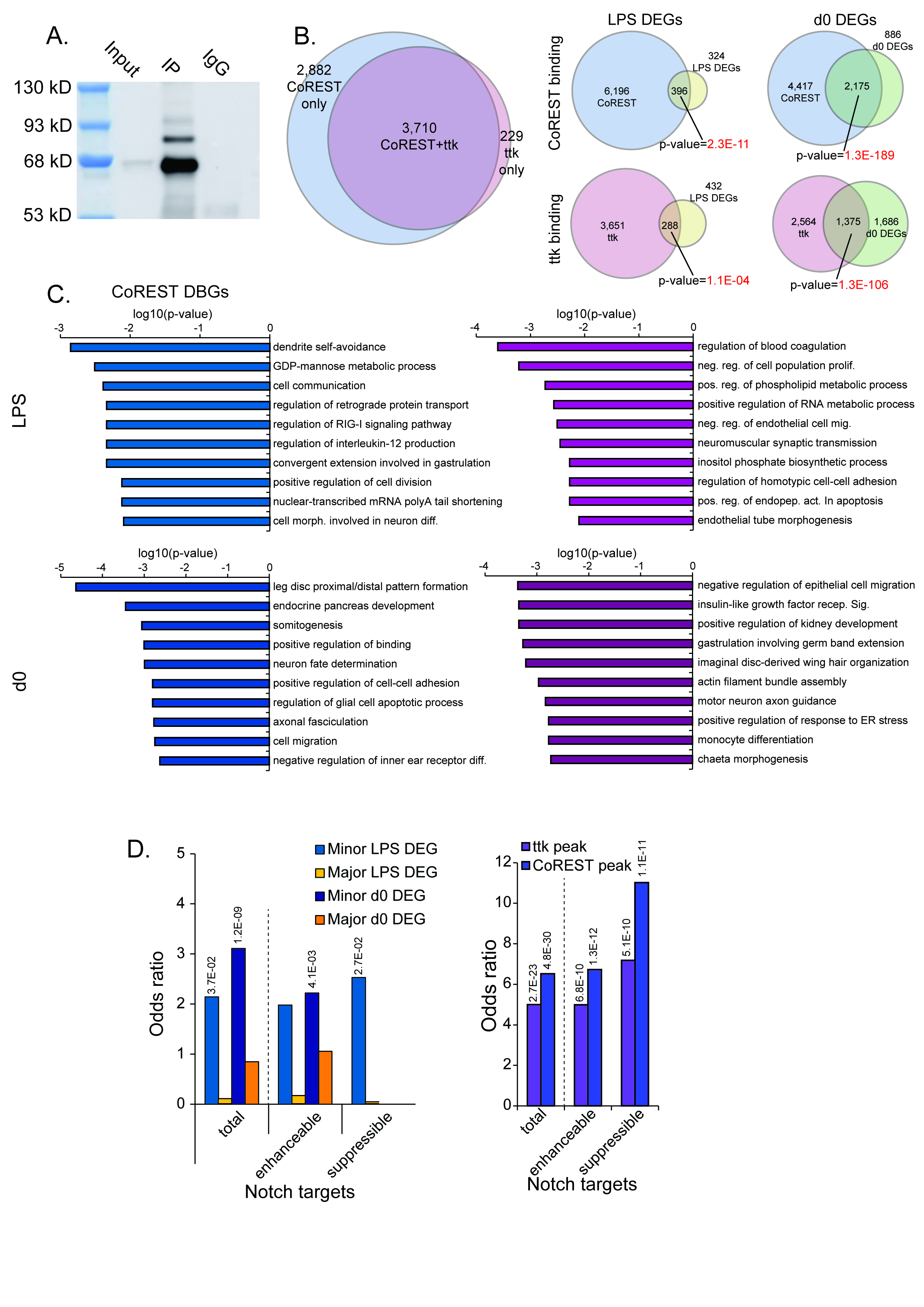

Supplement: S1 Fig — A) IP western blot of ttk using the custom antibody implemented in this study. Inputs represent 5% of IPs. B) left: similar to Fig 2D, but utilizing genes for overlaps instead of peaks, illustrating that the vast majority of genes marked with ttk are also marked with CoREST. Right: Venn diagrams showing overlap between DEGs seen between Majors and Minors at the late pupal stage (LPS) and d0 as compared to genes bound by either CoREST (top row) or tramtrack (bottom row) illustrating that genes bound by by either ttk or CoREST are significantly enriched for DEGs at both stages examined here. C) top 10 Gene Ontology (Biological Process) terms enriched among genes differentially bound (DBGs) by CoREST (left) or ttk (right) in the LPS (top row) or d0 (bottom row) as compared to genes bound by a non-differing peak in the same context. D) LPS and d0 DEGs biased to Minor workers are significantly associated with Notch signaling in fly but Major DEGs are not (top). Likewise, genes featuring a ttk or CoREST peak are significantly enriched for fly Notch target genes (bottom). Bars represent fishers exact test odds ratio, p-values above bars are from a fisher’s exact test. Results given for both all Notch targets as observed in fly, as well as those activated and repressed by Notch separately. (TIF) [file pgen.1009801.s001.tif]

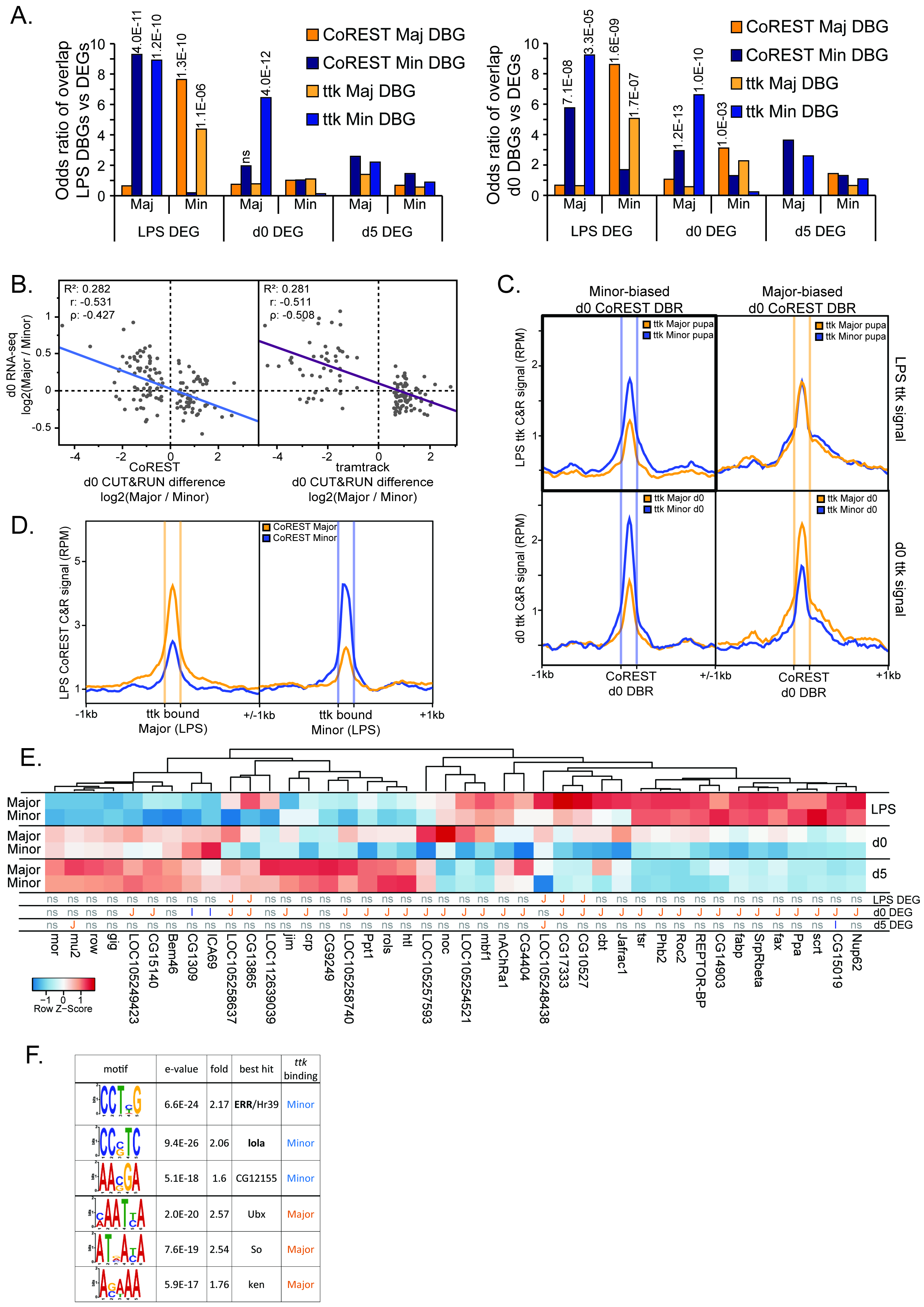

Supplement: S2 Fig — A) Fisher’s exact test odds ratios of overlap between differentially bound genes (DBGs) and differentially expressed genes (DEGs) for CoREST and ttk. Left: overlaps using DBGs in the LPS vs all DEGs presented here. Right: overlaps using d0 DBGs vs all DEGs presented here. Notably, ttk LPS binding shows far more significance of overlap with d0 DEGs as compared to the same analysis using CoREST DBGs. B) Scatterplots showing correlations between d0 caste brain differential expression (RNA-seq log2 Major/Minor) compared with CoREST (left panel) and ttk (right panel) d0 differences in binding between castes, as for Fig 3A but comparing d0 CUT&RUN differences to d0 RNA-seq differences. C) The reciprocal of Figs 3B and S2D, plotting ttk signal (RPM, replicate-averaged) for LPS (top row) and d0 (bottom row) at regions differentially bound (DBRs) by CoREST between d0 castes, illustrating that regions bound by CoREST more highly in d0 Minors shows Minor-biased enrichment of ttk in the LPS. D) Metaplots showing LPS CoREST signal for Major and Minor at regions differentially bound by ttk between LPS castes. The same regions as for Fig 3B are used but plotting LPS CoREST signal, illustrating that within-timepoint, CoREST shows the expected caste-biased enrichment at ttk caste-biased regions. E) heatmap as for Fig 3D but with late pupal stage included. F) Top three motifs derived from de novo motif enrichment run on either (top) Minor-biased or (bottom) Major biased ttk-peaks, compared to a background of all non-differing ttk peaks. Bolded best hits are those significantly biased in expression to the caste with the associated motif (ERR and lola). (TIF) [file pgen.1009801.s002.tif]
