# Supplementary material for: Tramtrack acts during late pupal development to direct ant caste identity
Source: PLoS Genet. 2021 Sep 22;17(9):e1009801. doi: 10.1371/journal.pgen.1009801 (PMC8489709; doi:10.1371/journal.pgen.1009801)
Supplement: Attachment — Submitted filename: reviewer_responses.v3.pdf [file pgen.1009801.s015.pdf]

REVIEWER 1:

In Camponotus floridanus worker ants, differences in caste behavior are programmed by epigenetic changes to gene expression driven by chromatin and transcriptional programs. For example, epigenetic factor coREST can reprogram caste behavior from soldier (major worker) to forager (minor worker). In this paper, Glastad et al. identify genome-wide transcriptional programs mediated by coREST and its binding partner ttk transcription factor complexes that differentiate minor versus major worker differences in caste behavior. The authors carry out cut and run experiments using antibodies against coREST and ttk in parallel with RNAseq from major and minor workers at late pupal stages and eclosion. They find that caste specific gene expression is apparent at pupal stages with genes involved in juvenile hormone metabolic process, and neuronal development and function as significantly different between major and minor workers. Expression of both ttk and coRESt is higher in minor caste and both genes associate with the promoters of genes associated major worker differentiation. The expression of coREST and ttk are inversely correlated with the expression of genes associated with major worker differentiation, suggesting their role in transcriptional repression of major worker genes in minor workers. Interestingly, they find that during late pupal stages, ttk is already bound at the promoters of major worker caste associated genes and coREST binding to the same sites occurs at eclosion. These suggest ttk likely primes or poises the major worker genes to be repressed in minor workers during pupal stages, and coREST binding locks in the transcriptional repressed state of major genes in minor workers to ensue transcriptional program. This is a great study unraveling the genome-wide gene regulatory programs, transcription and chromatin factors, and hormonal that contribute to differentiation of caste-specific behaviors. I think the paper is very well written and should be published in PLoS Genetics. Just a few minor comments below.

We thank the reviewer for their positive view of our work.

1- Fig1C. it seems like differences in jhe expression in minor versus major workers oscillates where LPS and d5 showing the biggest caste-specific difference whereas d0 and d10 shows very little. Can the authors elaborate on this with respect to the development (and critical periods) of caste specific behavior?

The reviewer is correct and we agree that these oscillations may reflect key periods of response to hormone JH3. Our view is that Jhe is highly variable in development because JH3 signaling is required in both castes at certain times, but then differentially required in a caste-specific manner at other times. We suggest that JH3 may be important at d0 for both castes and thus Jhe expression must 'unify' at this timepoint, but then again diverges at d5 for caste-specific JH3 requirements. We further propose that the level of Jhe in d10 is reflective of the final stable state in the organism, as this is the period after which HDACi-based reprogramming is no longer possible and there are very few caste differential genes in our RNA-seq data (Glastad et al., 2020, *Mol Cell*)

Because Jhe is not a focus of this study, we feel that elaboration on this point is outside the scope, so we have not added to the text on this point in the revised manuscript.

2- At the top of the GO terms shown in figures 1 and 2 are genes involved in R7 cell development and eye-antennal disc morphogenesis. Maybe I missed this but how do the authors think this gene cluster contributes to caste specific behaviors?

We thank the reviewer for pointing this out. While these GO terms may reflect true importance of optic system development in phenotypic differences between castes, we propose these terms may be partially due to the imprecise nature of GO terms in a diverged species such as ant, when leveraging functional inference from fly. That is, ttk and many target genes have been associated with regulating R7 cell fate in fly, however many of these genes have multiple neurodevelopmental functions. Because our GO analysis preferences specificity over generality, this term may reflect multiple neurodevelopmental genes for which this term is most specific, but possibly not the most accurate, in particular, given the well-studied nature of R7/R8 development relative to other neurodevelopmental models in fly. Indeed, in response to this point, we inspected the list of genes contributing to this term, finding many genes with multiple roles in neurodevelopment, not only development of the optic system. For example, the genes lola, chinmo, bab1, opa, and ss are all assigned this term, and while most are implicated in some form in fly eye development, most have multiple other roles in neurodevelopment or neuronal remodeling. Further, more specific eye TFs. such as so and lz, and are not differentially expressed between castes.

Nevertheless, we have now mentioned this in the results section (lines 89-92), and a corresponding column to Table S1.

3- In Fig 3D, the transcriptional profiles of major and minor workers at day 0 is quite different. However, by d5 they look more similar between the two castes. It would be good to elaborate on this as well.

The reviewer is correct, and this was a major component of our previous paper (Glastad et al., 2020, *Mol Cell*). We propose this is related to the formative age of d0 in ants (possibly equivalent to the first years of human development and neuronal pruning/remodeling) which becomes more 'ossified' by d5 and even more so by d10. This was a major motivating factor in examining the LPS-d0 timepoint, which we have now clarified in the Results section (lines 79-81).

4- Like 624 "normalized signal) of genes featuring Major-biased expression at d0 but pupal differential binding of tramtrack but..". is the first "but" supposed to be "by"?

We thank the reviewer for pointing this out. This point is an important observation of this study but complex to convey succinctly, thus we appreciate the reviewer noting its confusing phrasing. We have now rephrased (lines 485-492).

5- A very interesting question that arises from these studies is what biases the ttk DNA binding in minor workers. Is this induced in response to pheromone detection? And what are the chromatin differences at critical genes such as jhe at LPS and day 0 minor and major workers? I do understand these might be too big as experiments to be added to this study but can be mentioned briefly in the discussion.

We agree with the reviewer(s) that this is an important question arising from this work, and one we hope to answer in future publications. However we speculate that, given that Major and Minor worker gross phenotype diverges in the late larval stage, a prior signal related to this is upstream of ttk differential binding. We also know that an important component of Major Minor differences are JH3 levels (Glastad et al., 2020 *Mol Cell*) which has also been found in other social insects. Further, in several social insects and possibly *C. floridanus*, Ecdysone (20E) signaling is stronger in the non-foraging/worker caste. Both of these hormonal signals affect

activation and repression of specific genes via nuclear hormone receptors (Met and EcR among others), as well as downstream TFs which bind DNA via their own action and cofactors that modify binding. Based upon our motif analysis results we see evidence that in Minors multiple TFs related to hormonal signaling are localized to regions repressed by ttk-CoREST, suggesting hormonal signaling to be a central element of this mechanism. However, distinguishing between cofactors of ttk and targets of ttk repression (that is, genes that modify ttk binding specificity to workers vs genes that ttk is actively blocking via repression of binding sites) is complicated by the nature of both multiple hormone-related TFs. For example, one protein whose motif we see enriched among Minor-biased ttk peaks, ftz-f1, has been linked to JH3 signaling (Dubrovsky et al., 2011 PNAS) and is implicated in negative regulation of EcR, the canonical nuclear hormone receptor for 20E (flybase), in conjunction with Hr39, another motif found here (highly similar), however ftz-f1 has also been implicated in activating genes in response to 20E (Zhu et al., 2006, MCB). Usp is a member of the EcR heterodimer but also upregulated by JH3 in honey bee, has been suggested to bind JHs, and may, through this action, repress typical EcR targets when bound to JH3 (Maki, 2004, Biochemical and Biophysical Research Communications).

We have now elaborated in the discussion to address these complex points (lines 272-380).

<u>REVIEWER #2:</u>

Summary:

In the manuscript "Tramtrack acts during late pupal development to direct ant caste identity" the authors investigate the molecular mechanisms underlying the phenotypic and behavioral diversity that exists in the florida carpenter ant. They use the minor and major worker castes as a model system to globally assess gene expression and binding of coREST and ttk in both the late pupal stage (LPS) and early eclosion (d0). They show that during LPS and d0 there is a negative association between ttk & coREST co-binding and expression of minor and major differentially expressed genes resulting in caste specific gene expression. They also find that LPS differential binding of ttk poises coREST gene repression of a subset of major-biased genes during early eclosion in the minor. Additionally, they find that ttk differentially bound genes in minors are important for hormonal regulation, linking transcriptional mechanisms to known hormonal regulation of caste specification. Overall, the work makes an exciting mechanistic argument that is well-supported by the data and we found it fascinating and would like to learn more! The CUT&RUN data looks very good and it is great to see this working in the brain and in a less-studied organism. We suggest below revisions to analyses and the presentation of the paper that we hope will give the reader a broader context for understanding the work and make the data easier to follow.

We thank the reviewer for their comments and agree their suggestions are quite helpful for streamlining the delivery of this interesting finding.

General comments:

(1) *Additional background regarding this system is necessary as many readers who don't know much about this or other ants will be interested in the work --what are the behavioral differences between major workers and minor workers? Do they develop at the same rate? In terms of methods, how do the authors discriminate majors and minors as pupae or after eclosion? What is "LPS" in terms of days? How far apart in days are*

LPS and d0? Do major and minor pupae reach "LPS" at the same time? Is this a challenge for the experiments or interpretation of the results?

These are excellent points. Major and Minor workers do spend different amounts of time in the pupal stage, which could potentially introduce a developmental confound to studies comparing Major and Minor development. However, one of the advantages to the LPS is that it is defined relative to eclosion from the pupal case, wherein Major and Minor workers are developmentally synchronized. We chose 1 day prior to eclosion as this timepoint, which can be identified visually by the coloration of the pupa under the pupal case. As a secondary control, for every batch of LPS samples, we collected 2-6 extra pupae that were not dissected. Instead, these were evaluated the following day to ensure that for each batch of pupa selected as the LPS, selection was appropriately timed and that these extra pupa had indeed eclosed the following day (e.g. one batch did not meet this criteria and the resulting CUT&RUN experiment was discarded).

Thus, LPS is only 1-2 days separated from d0 samples, however in this time frame extensive molecular change occurs, likely concomitant with the newly eclosing ant encountering the outside world (nest environment). We have now added more detail outlining this as well as more specificity on sample selection, to the introduction (lines 59-60) and to the methods (lines 312-315). We also add more detail on the *C. floridanus* system to the introduction.

(2) *The authors find that many sites/genes are bound by both coREST and ttk (several thousand). Is this to be expected for these two types of transcription factors (i.e. chromatin remodelers and regulatory TFs)? Do the authors expect that all of these genes are related to caste specification?*

We thank the reviewer for pointing this out. While we did indeed find many sites bound (~8k peaks with CoREST, ttk, or CoREST+ttk), only a small subset (200-300 depending on stage or TF, < 5%) of these significantly differed between castes. While genome-wide ChIP-seq datasets of ttk in fly are limited, CoREST has been shown to bind 4-6k sites (Macinkovic et al, 2019 NAR) which is similar to our observation. Furthermore, given the prior observations of the interrelated function of CoREST and ttk (eg Dallman et al., 2004 J.Neurosci.) we feel that the result of ttk 'nesting' within CoREST targets to fit this model.

Because only a small subset of CoREST/ttk peaks appear to significantly differ between castes, we propose that many genes targeted by CoREST/ttk are repressed in a caste-independent manner, in line with the known neurodevelopmental roles of these proteins. Instead we argue that this system has been "repurposed" to regulate a small subset of targets in a caste-dependent manner, possibly in a celltype or stage-specific way. We have now added clarification of this to the results (lines 148-150) and discussion (lines 237-240) sections.

(3) *The authors focus much of their analysis on the idea that at LPS, tramtrack "poises" genes for later coREST binding at d0, and that this is predictive of d0 gene downregulation but only in Minor workers. But the larger story--that coREST and ttk are co-bound at most sites, and that they repress genes in both castes but different genes, is given less emphasis. Do the authors think that the co-bound sites were poised by tramtrack earlier in development (i.e. before the time points analyzed here)? What allows tramtrack and coREST to bind and repress different sites in the two castes, and how much overlap is there in their binding? It's reasonable for these questions to be addressed in a future publication, but it would be helpful in this paper if the authors*

could raise these questions and describe the extent to which they can or cannot answer them with the current datasets.

We agree that these are important questions. As with the previous comment from this reviewer above (#2) we have now added more clarity to the results and discussion to illustrate that the majority of sites *do not* differ between castes, and it is only a subset of sites that differ in a caste-specific manner (at least in these data from these timepoints). We feel that it is unlikely that the majority of non-differing sites seen here are simply reflective of regions that were caste-biased in a prior timepoint, instead it is likely that these are simply reflective of the conserved role of these factors in regulating neurodevelopment. We have now added more clarity to the discussion on this point (lines 240-243).

Regarding how ttk is targeted to the subset of genes it regulates in a caste-biased manner: this is also an important outstanding question to which we currently do not have a definitive answer. We believe that our motif analysis suggests a definite role of hormone-induced factors in this mechanism, however exactly which co-factors are directing this repression, and which (co-factor target sites) are the target of repression is currently unknown. This is complicated by the fact that multiple hormonal-signaling-related factors identified play roles in both JH3 and Ecdysone signaling, as noted in response to the reviewer's question #6 below. While we speculate that, given the preponderance of evidence suggesting that Minor worker transcriptomes are dominated by JH3 signaling, it is possible that Methoprene tolerant (Met) plays a role in targeting ttk to specific sites for repression in Minor, however, without empirical validation this remains unresolved.

Related to this, in Figure 3A, it appears there are similar numbers of peaks that are "tramtrack-only" in pupae in both majors and minors, but the distributions are quite different (both along the X axis and, as discussed, along the Y axis). What's up with the "tramtrack-only" sites in majors?

We would first like to clarify to the reviewer that what is presented in Figure 3A are genes differentially bound by CoREST or ttk, regardless of the presence of the alternative TF. We apologize if this was not clear and have now clarified this throughout.

Regardless, the reviewer makes a good point, and this is one that we did not have the statistical confidence to point out directly in the current MS: that the distribution of Major-biased ttk points are much less defined with respect to RNA-seq log2 fold changes (y-axis) as compared to Minor-biased ttk point/genes. This may further support a Minor-bias in the activity of ttk. For example, when comparing LPS CoREST to d0 RNA-seq differences (top left plot of 3A) the correlation is relatively weak, and this is evidenced by the 'clumped' points in 3A upper left; however, when comparing d0 CoREST to d0 RNA-seq differences (3A, bottom left) these become much more distributed in accordance with the stronger correlation between d0 CoREST and d0 gene expression. When performing the same comparison using ttk differences (right plots) there is a strong correlation between d0 expression differences and LPS ttk binding (3A, upper right) for Minor-biased ttk (correlating with Major-biased gene expression) but not for Major biased ttk (as compared to Minor-biased gene expression). Taken together, this suggests that the strongest impact of ttk on determining caste-based gene expression is in repressing genes in Minor workers (that are thereby Major-biased at the expression level). We have chosen not to added this point to text as it is both speculative and complex, however it is a subject we plan to follow up on in future publications.

(4) *Do the authors know whether coREST and ttk are differentially expressed at earlier developmental time points (lines 74-75)?*

We have not assayed this, however it is possible that the CoREST-ttk repressive axis acts throughout caste determination. Conversely it is possible that this mechanism is confined to the late stage of pupal development and early adulthood in accordance with behavioral differentiation, and that other mechanisms guide morphological differentiation between Majors and Minors in the late larval instars. We agree this is a worthwhile question that we will be exploring in future work.

(5) *The authors don't assess the function of any gene in this paper (we understand how difficult this is in ants), and therefore it's hard to know how crucial this poising is for minor worker behavior or caste identity, what sort of cellular complexity is involved in the neurons generating behavioral differences, etc. We suggest as a start that the authors could use the antibodies they have in hand to stain the brains of major and minor workers at the relevant time points. They could analyze how broadly tramtrack is expressed and whether differences in expression are broad and moderate in degree, versus whether tramtrack expression varies by caste much more but in only a subpopulation of cells.*

We agree with the reviewer that this is worth exploring. In response, we performed IF using our antibody to ttk in LPS Major and Minor brain sections (Reviewer Figure 1, below). While we did not see any high confidence localized differences in ttk between castes, broadly ttk does appear higher in Major workers here. However confident quantification would require optimization of permeabilization and staining, and given the time required for this we have provided the preliminary IF results for the reviewer but opted to not include this in our final manuscript. In further support we also performed western blot using brains of sample types used in this study, and found higher overall protein levels in Minor workers supporting our RNA-seq results (Reviewer Figure 2, below; because of individual animal variation, this would require many more samples). Nevertheless, the reviewer's suggestion is excellent and we will explore the distribution of ttk and CoREST in brains in future studies.

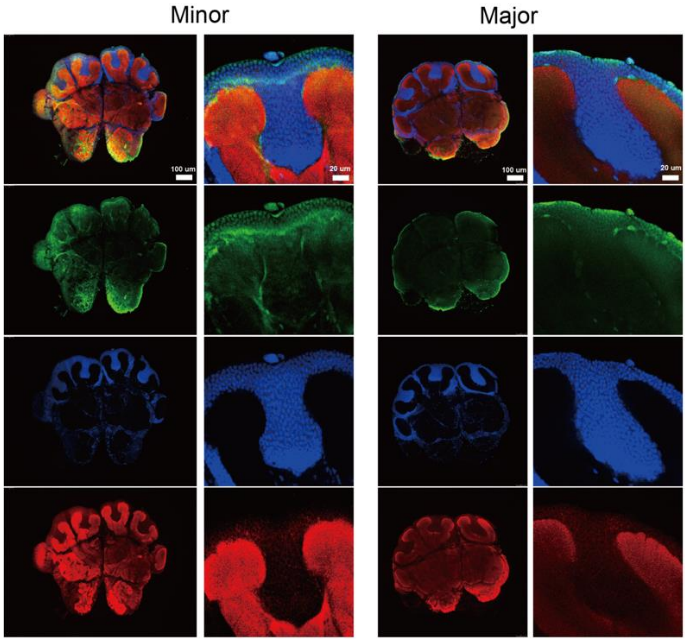

Reviewer Figure 1: Brain immunostaining for ttk in Minor (left) and Major (right) worker brains. C. floridanus brains were fixed in 4% paraformaldehyde in PBST (1xDPBS + 0.1% Tween-20) overnight at 4°C and washed in DPBS for 3 times before embedding in 4% agarose gel. 100µm cross-section were performed using Vibratome (Leica VT1000S), permeabilized in PBST (1xPBS + 0.5% Tween-20), and blocked in 5% goat serum in PBST (1xPBS + 0.1% Tween-20). Sections were incubated with the following primary antibodies diluted in blocking buffer overnight at 4°C: 1:20 mouse anti-Synapsin (3C11, DSHB); 1:700 custom rabbit anti-ttk. Samples were then washed 3x in DPBS, followed by incubation with secondary anti-mouse immunoglobulin G (IgG) (H+L), Alexa Fluor 568 and anti-rabbit IgG (H+L). Alex Fluor 488 (ThermoFisher) was 1:1000 diluted in DPBS and incubated with samples overnight at 4°C, followed by washing, and DAPI counterstain (2ug/ml in DPBS, Thermofisher). Default imaging parameters including “laser intensity” and “gain” and “offset” were calibrated using Minor condition.

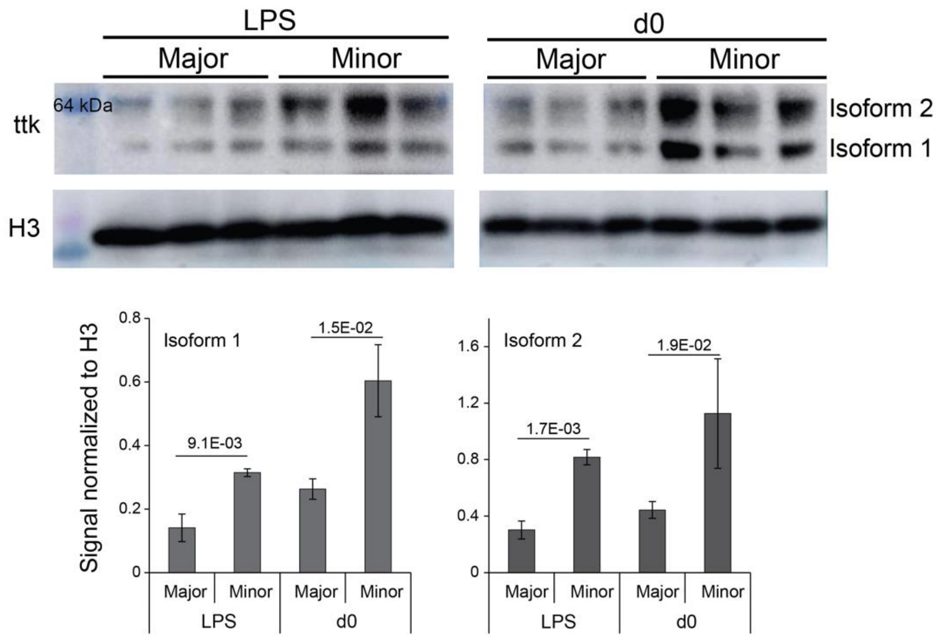

Reviewer Figure 2: western blot of ttk for LPS (left) and d0 (right) Major and Minor worker brains. Two isoforms of ttk are detected (vs one in IP western, isoform 2) but both show the expected pattern of higher levels in Minor workers at both timepoints.

(6) *In Figure 4, the authors show that many sites bound by tramtrack specifically in Minor workers are in genes that have functions related to hormone signaling. Is this a cause of differential hormone signaling in the two castes, or an effect of differential hormone signaling in the two castes? We ask because the hormone pathways represented by the four highlighted genes don't appear to be the JH and insulin pathways implicated in caste specification.*

We agree that the centrality of hormonal signaling to this mechanism is of great importance. In the current study we lack the information to properly discriminate between cause and consequence. However, given that gross phenotypic differentiation between Major and Minor workers occurs in the late larval stage, it is probable that ttk is downstream of signal(s) established earlier in development. Because ttk appears to be repressing sites that have motifs associated with multiple genes implicated in Ecdysone signaling, and that Ecdysone has been previously associated with non-foraging castes in social insects (Penick et al 2011, *Journal of Comparative Biology*; Robinson et al 1991, *Journal of Insect Phys*; Opachaloemphan et al 2021, *Genes&Dev*), and that we previously showed that Minors have high JH3 levels (Glastad et al 2020, *Mol Cell*), it appears that ttk may be repressing genes activated by Ecdysone in conjunction with activation of JH3 target genes via another mechanism. Indeed, Ftz-f1, Hr4, and usp, are known mediators of Ecdysone signaling in fly, thus the motifs most enriched among ttk-repressed regions (predominantly gene promoters) in the JH3-dominated caste (Minors) appear to be associated with genes downstream of the alternative hormone.

We realize this begs the question of what motifs are actually directing localization of ttk, which is of great interest to us as well. However multiple other motifs are enriched among both castes' ttk sites (lola, Kr, pad) which may be responsible for ttk localization for repression. Unfortunately, our data lack the resolution for statistically sound co-occurrence analysis, particularly in the context of using motifs from fly which may have diverged in the intervening

300my between ants and fly. However we are highly interested in addressing these questions in future work.

We have now added discussion of the cause and effect to the discussion (lines 283-286).

(7) *In GO analyses in Figures 1 and 2, the authors identify many eye-related genes. Our understanding is that whole brains were used in these chromatin and gene expression profiles, do eyes develop differently in the two castes? Could differential timing of eye development confound the analysis, or is it a critical caste-specific difference that causes caste-specific differences in behavior? In future studies, it might be fruitful to separate optic lobes and central brains.*

We agree with the reviewer (and reviewer 1) that this is an important point. For a complete response, please refer to reviewer 1, point 2 above.

(8) *We also suggest a comparison to sex differentiation as another model of how the same genome can be used to generate two "morphs," especially as insect sex determination also relies on a BTB transcription factor (Fruitless) hypothesized to specify one morph by suppressing transcription of genes used in the other. (https://journals.plos.org/plosgenetics/article?id=10.1371/journal.pgen.1009338)*

We agree with the reviewer that this is an important contrast to be included in future studies. Sex differentiation is indeed an excellent alternative pathway of phenotypic plasticity, and indeed several genes known to play roles in sex differentiation also show caste-specific expression (Klein et al., 2016, *PloS Genetics*). However, because *C. floridanus* males cannot be easily produced in the lab and are only available during limited times of the year in the native range of *C. floridanus*, we are unable to assess this at this time. However this is an outstanding point for consideration in future studies.

However the reviewer's suggestion and included reference is an excellent parallel between these systems, and we have added this to the discussion (lines 225-229).

Specific comments on the writing:

We think these changes could improve the manuscript, but if the authors disagree they are free to ignore them:

(1) *We suggest removing jargon and abbreviations when possible.*

We have endeavored to remove jargon and abbreviations when possible.

(2) *Line 15-17: The circuits that are in between genes and behavior are elided. If the authors are arguing that there are no anatomic (wiring) differences between the castes, and that caste is entirely controlled by differential gene expression in neurons of circuits that are wired identically, they should say so explicitly.*

We have now changed this in response to the reviewers important point (line 16). We were not arguing that neuronal wiring is not important in this mechanism, and is doubtlessly central to it.

(3) *Line 39: The use of the word plasticity is confusing as it could mean developmental plasticity, behavioral plasticity, neuronal/homeostatic plasticity... Please clarify.*

We have added clarification (line 39).

(4) *Line 57: Probably "hormone production" rather than "hormone expression."*

We have changed this.

(5) *Line 198: "Epigenetic programming mechanisms." We suggest using more precise language, as many meanings have been tacked onto the word epigenetic. (See https://www.nature.com/articles/nrg.2017.32).*

We agree on this point and have changed this wording for more precision.

Specific comments on figures and figure legends:

Figure 1:
Needs more detail in the legend so we know what we are looking at. For example (B) Volcano plot of DEGs: What was sequenced?
We have added more detail to the figure legends here and throughout.

Fig 2:

- *Fig 2A clearly shows the negative association between gene expression and binding of ttk and coREST, however Fig 2B is less clear and rotating it to resemble the orientation of Fig 2A would be helpful. In Figure 2A, were non-significant dots removed? How about including all the dots and showing ones with significant changes in a different color from unchanged ones?*

We have now altered figure 2A and B to increase ease of interpretation. We have also included all genes with non-significant differences in binding (that still possess a valid peak) in light gray.

- *Fig 2C very clearly shows example genes where coREST and ttk are binding in a caste-specific way to allow differential gene expression. What stage is this data from? Please add scales to X and Y axes here and throughout. The authors are showing here and in Figure 2A that ttk and coREST can bind different sites in the two castes. How?*

We have now added data ranges to each track, and a label for the stage the data were taken from (late pupal stage), and apologize for the oversight.

As for the mechanism of differential binding, we do not currently know but it is an area of active interest for us. It is very likely that ttk is a component of larger multiprotein functional complexes in which ttk may provide specificity, along with other partner TFs, for specific genes.

In our motif analysis of differentially bound regions where we compared Major or Minor DBRs to all non-differing ttk peaks (Fig 4B, and new figure S2C), we see different motifs enriched among Major and Minor peaks suggesting either ttk is repressing genes that are targets of these TFs, or these motifs represent TFs in complex with ttk and CoREST. Distinguishing between these two alternatives with our current data is difficult, which is why we chose not to make many definitive conclusions regarding the hormonal signaling motifs we saw enriched among Minor-biased ttk peaks. This is further complicated by the fact that several of the TFs detected among these peaks have been implicated in JH3 as well as 20E signaling. For example, ftz-f1 has been linked to JH3 signaling (Dubrovsky et al., 2011 *PNAS*) and is implicated in negative regulation of EcR, the canonical nuclear hormone receptor for 20E (flybase), in conjunction with Hr39, another motif found here (highly similar). Usp is a member of the EcR heterodimer but also upregulated by JH3 in honey bee, has been suggested to bind JHs, and may, through this action, repress typical EcR targets when bound to JH3 (Maki, 2004, *BBRC*).

Given the highly complex nature of hormonal transcriptional regulation illustrated above and the fact that few of these TFs are differentially expressed at the mRNA level between castes (ftz-f1 is higher in Minor) it is difficult to provide more definitive conclusions. Because we have evidence here and elsewhere (Glastad et al., 2020, *Mol Cell*) as well as in other social insects, that workers/foragers show higher JH3 signaling, and non-foraging castes higher 20E, we believe that with these data the most definitive statement we can make is that ttk is central to regulating or transducing these important hormonal processes; however exactly how ttk is localized to these genes, and whether an identified motif is directly indicative of a co-factor or a target for repression we do not believe we can definitively conclude. However we are very interested in this exact question and will seek to address in future studies. As it stands, we have added more to the discussion on this point (lines 272-380).

- *Figure 2D: What stage is this? Do the authors find a motif in the ttk-only sites? Is there any difference in motifs in ttk+/coREST- and ttk+/coREST+ sites? Here and in supplemental Figure 1, the use of Venn diagrams is confusing. How about upset plots instead? They are designed for this type of multi-factor comparison.*

Motif enrichment was performed on peaks called from merged data from all samples. We have now added more clarity to the respective methods sections (lines 379-380 and 401-404).

The primary motif in both sets (ttk+/CoREST- and ttk+/CoREST+) are highly similar, being dominated by stretches of C/G residues. Based upon central enrichment among binding sites (a good way to break motif 'ties' and evaluate centrality to a given binding factor) for ttk+/CoREST- peaks:

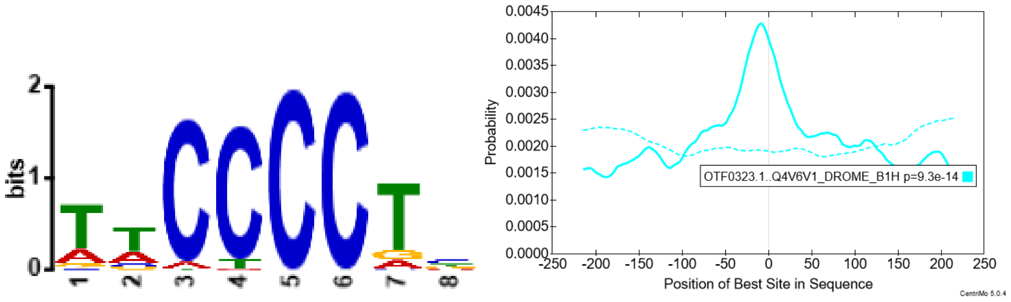

This is highly-similar to that found in Fig 2D, as well one of the determined fly ttk motifs. Thus it does not appear that CoREST presence influences the binding motif of ttk.

We have now split figure S1B into separate venn diagrams for each overlap to improve readability.

Fig 3:

- *Figure 3A: There aren't many dots along zero here, but there are some. Were non-significant dots filtered out, or is this because tramtrack-differential peaks at LPS were the only ones included in this plot, and those peaks tend not to fall at 0 in these other analyses either?*

For the x-axis (CUT&RUN binding differences) we cannot see any points at zero, which is consistent with the fact that we are only presenting significant differentially-bound genes. For the y-axis (log2 fold change between Major/Minor RNA-seq) there are points at or near zero as these were not filtered on differential expression.

- *Figure 3B: We were not able to understand this figure.*

We agree with the reviewer that this was confusing. We have changed this panel to show d0 CoREST signal at LPS pupal differentially-bound regions, and limited presented comparisons to only this comparison. We moved the previous panels to the supplement to keep this recpirocal comparison in the manuscript in some form. We have updated the results section text (lines 175-178) and figure panel accordingly.

- *Figure 3C: The legend is unclear; on line 625 it says that "ttk is poising minor biased genes for repression prior to eclosion" however in the diagram of Fig, 5 ttk is poising major biased genes.*

This was a typo and we thank the reviewer for catching it. We have changed the legend for this panel for clarity.

Fig 5:

- *Figure 5 is great*

We strongly agree with the reviewer!

<u>REVIEWER #3:</u>

Ants are eusocial, within an ant colony castes divide reproductive labor. After mating, the queen spends the rest of her life reproducing and the workers of the colony forego reproduction in exchange for carrying out other colony tasks including nursing, foraging, and defense. In some species, the worker caste has been expanded to include minor and major workers, which are optimized to carry out subsets of the general non-reproductive tasks. In the study species of this paper, the ant Camponotus floridanus, there are small minor workers that nurse young and forage, and large major workers that defend the colony. Regardless of caste, these individuals, who can differ in morphology, reproduction, and behavior, are highly related and thus their trait differences are due to epigenetic rather than genetic causes. This makes C. floridanus a great system to understand the epigenetic basis of behavioral variation and how epigenetic processes interact with hormonal processes, as hormonal pathways, and their differential regulation, are known to be critical during the development of ant castes. Several past works by Glastad et al. have made critical insights into the importance of epigenetic regulation in social insect caste formation and behavioral differences. In particular, a landmark study by Glastad et al (2020; Molecular Cell) found that a key mediator of histone/chromatin dynamics and transcriptional regulation, CoREST, is critical for behavioral reprogramming of minor/major castes in C. floridanus. In this current work, Glastad & Berger build on their recent work by describing the transcriptomic signatures of a key phase during the pupal-adult transition in minor and major workers, suggested to be a transformative period in setting the stage for minor and major worker caste-specific behavioral programs. The most significant finding of this work is that the protein Tramtrak (ttk), known to interact and function with CoREST, both aides CoREST in establishing minor-major behavioral programs and surprisingly may precede CoREST function and poise/prime genes (that are differentially expressed between minors and majors) for subsequent CoREST activity. The authors found many ttk+CoREST bound genomic regions as well as ttk-specific regions, all of which will be of great interest for future investigation. While I think this work will provide an excellent resource for future work towards understanding how

minor and major workers establish their respective behavioral programs, I have a couple major comments (along with some minor comments) that I think the authors should address.

We thank the reviewer for their assessment and comments.

Major Comments:

(1) In several parts of the paper, the authors jump to conclusions regarding the role of ttk in the 'ttk-CoREST repressive axis' that governs caste-specific behavioral programming. While their previous work used pharmacological and RNAi experiments to implicate CoREST and CoREST-histone dynamics in worker caste behavioral reprogramming, this paper extends causative implication of ttk in a way that would require functional experiments. For example(s), the authors say: "Here, we show this pathway is engaged naturally on a large genomic scale during late pupal development to regulate multiple genes to program Minor behavior, via key function of tramtrak (ttk), a DNA binding partner of CoREST" and "Our results indicate that ttk drives the function of CoREST in C. floridanus caste-determination, where ttk directs CoREST binding to a specific subset of loci for caste-biased repression", and "In conclusion our results elucidate a repressive axis that governs caste identity in C. floridanus worker ants". These statements (and other similar phrases/wordings) either need to be modified to reflect the correlational aspect of this work, ***or*** *the authors need to consider doing loss-of-function experiments involving ttk in order to support their claims as they are currently worded. For instance, following the knockdown of ttk, are 'adult major-biased genes' upregulated at eclosion (like Jhe) and what happens to CoREST function (i.e.: demonstrate the 'poising' function of ttk on subsequent CoREST activity)?*

We agree with the reviewer that such statements were made too strongly and we have changed these statements throughout the manuscript.

Related to this, in lines 142-151, the authors describe correlational data regarding Notch yet unlike other result paragraphs of the paper, they do not end the paragraph with any conclusions about the data. The authors should either remove that data or make explicit whether you are just suggesting Notch signaling to be relevant to ttk activity, in this context, or explicitly demonstrating this. While the authors do state in the discussion that the Notch data is speculative, they still include Notch at the end of their paper in the following sentence: "high degree of conservation in several of the observed systems here—CoREST, Notch, and nuclear hormone receptor binding—our findings shed light on how the hormone-epigenome axis effects long-lasting behavioral plasticity across the animal kingdom". I would remove Notch from this conservation sentence and focus on the other more supported elements in the paper.

We agree with the reviewer and we removed the mention of Notch from this section of the discussion, and added a conclusion sentence to the aforementioned results section to reflect the correlational nature of these results. We agree with the reviewer that, without explicit molecular demonstration we should be wary of strong statements; however, we believe that—given prior observations of Notch and caste, notch and ttk, along with our observation of Notch, ttk, and caste—including these data in this paper will be of utility to the field.

(2) Lines 41-44: The authors discuss the relevance of chromatin modifications with plasticity in eusocial insects. Unfortunately, this section serves as more of a strawman-type point, rather than emphasizing the known literature regarding other mechanisms (DNA methylation) that are complementary to the ones the authors focus on in this study (histone modifications). It comes off

as an attempt to say histone modifications are the only form of epigenetic regulation that is (so far) deemed important to eusocial insect biology. This is either done to emphasize the novelty/impact of the authors' new work (if so, unnecessary as this is important work), or due to poor scholarship. There is no need to downplay DNA methylation, the authors have done incredible work to demonstrate the importance of histone modification in eusocial insects; these two mechanisms do not need to be made mutually exclusive in significance, relevance, etc. I am not going to write here an entire review perspective on how DNA methylation has been established to be important for eusocial insect plasticity (including ants; the authors should not just rephrase this section to just downplay DNA methylation in ants either). Still, I will highlight several landmark papers that show DNA methylation is important: (1) Kucharski et al. (Science, 2008) functionally demonstrates for the first time that DNA methylation is critical in caste determination in honey bees; (2) Lyko et al. (PLoS Biology, 2010) demonstrate the caste-specific methylomic profile of the brain and associate it with alternative-splicing in social insects, and follow this up with (3) Foret et al. PNAS 2012 (further supported by Flores et al BMC Genomics, 2012); (4) Biyarlay et al. (PNAS, 2013), definitively demonstrated the role of DNA methylation and alternative splicing regulation in honey bee fat body tissue, targets of which overlaps with parallel contexts (nutrient-deprivation, forager versus nurse behavior, queen inhibitory pheromone environment, and developmental knockdown of DNA methylation) where overlapping differentially methylated genes were found; (5) Biergans et al (PLoS ONE, 2012) demonstrated that DNA methylation is important for olfaction-related associative long-term memory in honeybees; (6) Herb et al., (Nature Neuroscience, 2012) demonstrated reversible epigenetic states associated with honeybee behavioral castes; (7) Bonasio et al. (Science, 2010) showed that C.floridanus larvae and adult workers have significantly more global-levels of DNA methylation than H. saltator, suggesting that species with higher caste complexity may employ DNA methylation to enable the generation of such complexity; (8) Bonasio et al (Current Biology, 2012) show that C. floridanus minor workers have higher levels of DNA methylation than major workers and queens, and that minor and major workers have genes that are differentially methylated in the adult and finally that DNA methylation is associated with genomic imprinting and alternative splicing; (9) Alvarado et al (Nature Communications, 2015) showed that C.floridanus minor and major worker larvae are differentially methylated on a global level and gene-specific level and pharmacological manipulation of DNA methylation during development can effect intra-caste sizing, levels of gene-specific expression, and methylation; (10) Demethylation in bumblebees leads to caste-specific aggression and reproductive phenotypes (Amarasinghe et al., Proc. R. Soc. B, 2014); (11) Glastad et al., (2016, Scientific Reports) demonstrated the widespread nature of caste-specific DNA methylation in termites, particularly as related to alternative splicing. The list goes on, and makes crystal clear there are multiple roles for caste differential DNA methylation in eusocial insects. The authors instead put all of the weight of their conclusion that DNA methylation's role being 'unclear' to one single paper (Libbrecht et al., Current Biology, 2016), a paper which not only completely overlooks the implications of all empirical/experimental studies done in social insects regarding the role of DNA methylation − hiding these references in the supplement, giving them zero weight, and focusing their work exclusively on biostatistical analysis of –omic data interpretation, but also extrapolates their findings, done in an ant where all individuals are literal clones (the clonal raider ant), where one would in fact predict it would be the least plastic and the least expected/predicted to require differential epigenetic regulation (like DNA methylation) in the first place.

This section needs to be heavily revised or just completely removed. Either focus exclusively on histone modifications or give proper credit to DNA methylation studies. The social insect community anxiously awaits the papers coming out of the Berger lab, as it has become an authority in this field over the last decade, so perpetuating this poor scholarship can have significant negative consequences in the trajectory of this community.

We appreciate the reviewer's attention to detail on this topic and we did not mean to dismiss other forms of epigenetic information, moreover, the reviewer's point is well made. Because we are not speaking to DNA methylation here, instead of a longer discussion of these important considerations brought by the reviewer and other work, we have simply removed the line about DNA methylation from the introduction.

Minor Comments:

Lines 67-68: 'acts first in pupal'--->acts first in the pupal...

Changed

Lines 94: "and correlates with queen development [39]". The Amdam group has experimentally/functionally manipulated the insulin substrate, chico, in this paper and showed that it regulates caste development in a causative (not correlational) way. Also, please refer to Mutti et al., 2011 in the Journal of Experimental Biology (also the Amdam group).

Edited accordingly.

Line 163: 'previous' should perhaps be 'preceding' instead.

Changed

Line 254: 'homones'---> hormones

Changed

Line 256: 'caste identity'--->behavioral caste identity or caste-specific behavior.

Changed

Line 257: 'to realize caste fate'---> to realize caste-specific behavior. "

Changed
