# Supplementary material for: Tramtrack acts during late pupal development to direct ant caste identity
Source: PLoS Genet. 2021 Sep 22;17(9):e1009801. doi: 10.1371/journal.pgen.1009801 (PMC8489709; doi:10.1371/journal.pgen.1009801)
Supplement: Attachment — Submitted filename: GlastadReview.docx [file pgen.1009801.s014.docx]

**Summary:**

In the manuscript “Tramtrack acts during late pupal development to direct ant caste identity” the authors investigate the molecular mechanisms underlying the phenotypic and behavioral diversity that exists in the florida carpenter ant. They use the minor and major worker castes as a model system to globally assess gene expression and binding of coREST and ttk in both the late pupal stage (LPS) and early eclosion (d0). They show that during LPS and d0 there is a negative association between ttk & coREST co-binding and expression of minor and major differentially expressed genes resulting in caste specific gene expression. They also find that LPS differential binding of ttk poises coREST gene repression of a subset of major-biased genes during early eclosion in the minor. Additionally, they find that ttk differentially bound genes in minors are important for hormonal regulation, linking transcriptional mechanisms to known hormonal regulation of caste specification. Overall, the work makes an exciting mechanistic argument that is well-supported by the data and we found it fascinating and would like to learn more! The CUT&RUN data looks very good and it is great to see this working in the brain and in a less-studied organism. We suggest below revisions to analyses and the presentation of the paper that we hope will give the reader a broader context for understanding the work and make the data easier to follow.

**General comments:**

(1) Additional background regarding this system is necessary as many readers who don’t know much about this or other ants will be interested in the work --what are the behavioral differences between major workers and minor workers? Do they develop at the same rate? In terms of methods, how do the authors discriminate majors and minors as pupae or after eclosion? What is “LPS” in terms of days? How far apart in days are LPS and d0? Do major and minor pupae reach “LPS” at the same time? Is this a challenge for the experiments or interpretation of the results?
(2) The authors find that *many* sites/genes are bound by both coREST and ttk (several thousand). Is this to be expected for these two types of transcription factors (i.e. chromatin remodelers and regulatory TFs)? Do the authors expect that all of these genes are related to caste specification?
(3) The authors focus much of their analysis on the idea that at LPS, tramtrack “poises” genes for later coREST binding at d0, and that this is predictive of d0 gene downregulation but only in Minor workers. But the larger story--that coREST and ttk are co-bound at most sites, and that they repress genes in *both* castes but *different* genes, is given less emphasis. Do the authors think that the co-bound sites were poised by tramtrack earlier in development (i.e. before the time points analyzed here)? What allows tramtrack and coREST to bind and repress different sites in the two castes, and how much overlap is there in their binding? It’s reasonable for these questions to be addressed in a future publication, but it would be helpful in this paper if the authors could raise these questions and describe the extent to which they can or cannot answer them with the current datasets.

Related to this, in Figure 3A, it appears there are similar numbers of peaks that are “tramtrack-only” in pupae in both majors and minors, but the distributions are quite different (both along the X axis and, as discussed, along the Y axis). What’s up with the “tramtrack-only” sites in majors?

(4) Do the authors know whether coREST and ttk are differentially expressed at earlier developmental time points (lines 74-75)?
(5) The authors don't assess the function of any gene in this paper (we understand how difficult this is in ants), and therefore it's hard to know how crucial this poising is for minor worker behavior or caste identity, what sort of cellular complexity is involved in the neurons generating behavioral differences, etc. We suggest as a start that the authors could use the antibodies they have in hand to stain the brains of major and minor workers at the relevant time points. They could analyze how broadly tramtrack is expressed and whether differences in expression are broad and moderate in degree, versus whether tramtrack expression varies by caste much more but in only a subpopulation of cells.
(6) In Figure 4, the authors show that many sites bound by tramtrack specifically in Minor workers are in genes that have functions related to hormone signaling. Is this a cause of differential hormone signaling in the two castes, or an effect of differential hormone signaling in the two castes? We ask because the hormone pathways represented by the four highlighted genes don’t appear to be the JH and insulin pathways implicated in caste specification.
(7) In GO analyses in Figures 1 and 2, the authors identify many eye-related genes. Our understanding is that whole brains were used in these chromatin and gene expression profiles, do eyes develop differently in the two castes? Could differential timing of eye development confound the analysis, or is it a critical caste-specific difference that causes caste-specific differences in behavior? In future studies, it might be fruitful to separate optic lobes and central brains.
(8) We also suggest a comparison to sex differentiation as another model of how the same genome can be used to generate two “morphs,” especially as insect sex determination also relies on a BTB transcription factor (Fruitless) hypothesized to specify one morph by suppressing transcription of genes used in the other. (https://journals.plos.org/plosgenetics/article?id=10.1371/journal.pgen.1009338)

**Specific comments on the writing:**

We think these changes could improve the manuscript, but if the authors disagree they are free to ignore them:

(1) We suggest removing jargon and abbreviations when possible.
(2) Line 15-17: The circuits that are in between genes and behavior are elided. If the authors are arguing that there are no anatomic (wiring) differences between the castes, and that caste is entirely controlled by differential gene expression in neurons of circuits that are wired identically, they should say so explicitly.
(3) Line 39: The use of the word plasticity is confusing as it could mean developmental plasticity, behavioral plasticity, neuronal/homeostatic plasticity... Please clarify.
(4) Line 57: Probably “hormone production” rather than “hormone expression.”
(5) Line 198: “Epigenetic programming mechanisms.” We suggest using more precise language, as many meanings have been tacked onto the word epigenetic. (See <https://www.nature.com/articles/nrg.2017.32> )

**Specific comments on figures and figure legends:**

Figure 1:

Needs more detail in the legend so we know what we are looking at. For example *(B) Volcano plot of DEGs*: What was sequenced?

Fig 2:

- Fig 2A clearly shows the negative association between gene expression and binding of ttk and coREST, however Fig 2B is less clear and rotating it to resemble the orientation of Fig 2A would be helpful. In Figure 2A, were non-significant dots removed? How about including all the dots and showing ones with significant changes in a different color from unchanged ones?
- Fig 2C very clearly shows example genes where coREST and ttk are binding in a caste-specific way to allow differential gene expression. What stage is this data from? Please add scales to X and Y axes here and throughout. The authors are showing here and in Figure 2A that ttk and coREST can bind different sites in the two castes. How?
- Figure 2D: What stage is this? Do the authors find a motif in the ttk-only sites? Is there any difference in motifs in ttk+/coREST- and ttk+/coREST+ sites? Here and in supplemental Figure 1, the use of Venn diagrams is confusing. How about upset plots instead? They are designed for this type of multi-factor comparison.

Fig 3:

- Figure 3A: There aren’t many dots along zero here, but there are some. Were non-significant dots filtered out, or is this because tramtrack-differential peaks at LPS were the only ones included in this plot, and those peaks tend not to fall at 0 in these other analyses either?
- Figure 3B: We were not able to understand this figure.
- Figure 3C: The legend is unclear; on line 625 it says that “ttk is poising minor biased genes for repression prior to eclosion” however in the diagram of Fig, 5 ttk is poising major biased genes.

Fig 5:

- Figure 5 is great
